# A lightweight mixup-based short texts clustering for contrastive learning

**DOI:** 10.3389/fncom.2023.1334748

**Published:** 2024-01-11

**Authors:** Qiang Xu, HaiBo Zan, ShengWei Ji

**Affiliations:** School of Artificial Intelligence and Big Data, Hefei University, Hefei, Anhui, China

**Keywords:** overlapping, text clustering, contrastive learning, data augmentation, mixup

## Abstract

Traditional text clustering based on distance struggles to distinguish between overlapping representations in medical data. By incorporating contrastive learning, the feature space can be optimized and applies mixup implicitly during the data augmentation phase to reduce computational burden. Medical case text is prevalent in everyday life, and clustering is a fundamental method of identifying major categories of conditions within vast amounts of unlabeled text. Learning meaningful clustering scores in data relating to rare diseases is difficult due to their unique sparsity. To address this issue, we propose a contrastive clustering method based on mixup, which involves selecting a small batch of data to simulate the experimental environment of rare diseases. The contrastive learning module optimizes the feature space based on the fact that positive pairs share negative samples, and clustering is employed to group data with comparable semantic features. The module mitigates the issue of overlap in data, whilst mixup generates cost-effective virtual features, resulting in superior experiment scores even when using small batch data and reducing resource usage and time overhead. Our suggested technique has acquired cutting-edge outcomes and embodies a favorable strategy for unmonitored text clustering.

## 1 Introduction

Medical records have been a key research focus in recent years (Campbell et al., 2007; Liu et al., 2019a) due to their invaluable insights into the developmental relationship between diseases and effective treatment options. However, semi-structured diagnostic cases or text reports typically constitute the bulk of patient information in the majority of these records, making it imperative to extract semantic information for selecting appropriate treatment plans and generating comprehensive patient follow-up reports.

Prior research efforts have primarily concentrated on specific aspects of medical records, such as that conducted by Lyu and Haque (2018), has focussed on early cancer detection, whilst Vieira et al. (2017) developed predictive models for diagnosing pathology. However, the fragmented and piecemeal nature of the data suggests that data miners may not meticulously consider the intricacies of medical diagnostic decision support. Therefore, as an effective data mining technique, clustering (Xu and Wunsch, 2005) plays an important role in the field of text analysis and semantic understanding. Clustering can be used independently to structure individual pieces of information or as a precursor to downstream tasks like classification. Among the many clustering algorithms, K-means (Hartigan and Wong, 1979), DBscan (Ester et al., 1996), hierarchical clustering (Murtagh and Contreras, 2012), and Gaussian Mixture Model (GMM) (Dempster et al., 1977) are the mainstream methods used for categorizing text based on the distance between sample points in the feature space. However, the high-dimensional sparsity of short texts often makes it difficult to reflect the similarity between datasets, and the learned representations are distributed in a narrow cone, resulting in unsatisfactory clustering results. Recent studies have shown that adding contrastive learning (Wu et al., 2018) to the clustering process can optimize the distance of the initial feature space, making the local data more compact and the overall features more uniform.

Contrastive learning has shown impressive outcomes in unsupervised sentence representation learning (Tang et al., 2022; Wei et al., 2022). The fundamental concept entails generating positive pairs and negative pairs via data augmentation (Wei and Zou, 2019), and feeding these pairs into a pre-trained model to minimize the distance between positive pairs while maximizing the distance between negative pairs. This optimization process aims to enhance sentence embedding. SimCLR (Chen et al., 2020) is one of the representative works in this field and experiments have shown that incorporating a large number of negative samples can improve the experimental results. Increasing the batch size is the simplest way to achieve this, but MoCo (He et al., 2020) proposed a method that utilizes a queue to store past small batches of datasets, thereby increasing the availability of negative samples. Moreover, momentum was employed to update the encoder in the queue, facilitating gradual updates and ensuring consistency in the feature representation of the data in the queue. However, this approach may result in longer model training time and increased computing power consumption during data transmission and parallel computing with GPUs. Research has confirmed that these issues stem from two primary factors. Firstly, insufficient contrastive samples, as positive and negative samples must be selected from each batch for use in contrastive learning. A small batch size may result in an inadequate number of comparison samples, limiting the amount of information learned and adversely affecting model performance. Secondly, the use of a queue to store past mini-batches of data necessitates comparing each mini-batch with the data in the queue, requiring additional iterations to complete the training process. In domains such as electronic platform shopping, stock trading, and catering, systems often divide order tasks into multiple small batches to reduce costs, save time, and ensure accurate processing. Therefore, how to optimize the model training time and memory consumption without affecting the clustering effect is a potential research direction.

**FIGURE 1:**
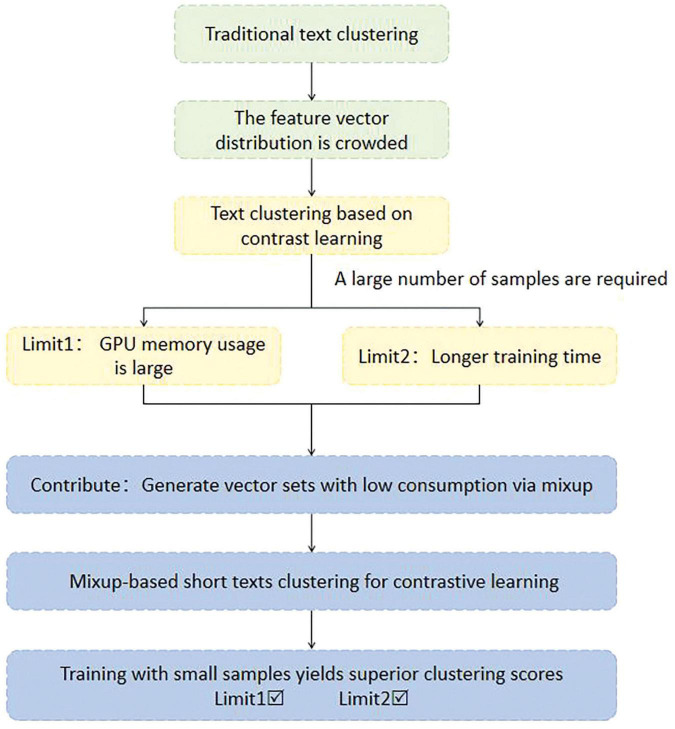
Relationship between motivation and contribution in this paper.

Given the aforementioned issues, this paper addresses the challenges by introducing mixup (Zhang et al., 2017) in a low-resource setting. The technique applies linear interpolation to the embeddings of positive pairs in the augmented dataset, generating an additional set of feature vectors to augment contrastive samples with low resource consumption. This feature vector set takes into account the semantics of the text and provides a set of virtual feature vectors in the representation layer that are closer to the original dataset, and minimizes the impact of deleting or inserting words on the semantics without significantly increasing the computational cost. This set of features is then fed into contrastive learning and clustering modules. Consequently, the model can learn more nuanced representations by controlling the weights, achieving a significant improvement in clustering scores within a mini-batch environment, even without loading additional text data. The constructed set of virtual vectors is stored in memory, enabling the direct reading of data during training and avoiding significant additional memory overhead. Therefore, this paper combines mixup and SCCL (Zhang et al., 2021), as shown in Figure 1, and proposes A lightweight Mixup-based Short Texts Clustering For Contrastive Learning (MCC). Eight datasets are used to evaluate the performance of the proposed MCC in short text clustering tasks.

For example, suppose many records mention symptoms such as fever, cough, and sore throat. These records can be clustered together, indicating a potential category of respiratory diseases, such as colds or flu. However, a challenge arises when dealing with rare diseases that are sparsely represented in the dataset. Consider a scenario where there are only a few records that mention a particular set of symptoms that are indicative of a rare genetic disorder. In traditional clustering, these records may not be effectively grouped due to their low occurrence and dissimilarity to other records. To address this challenge, the proposed contrastive clustering method based on confusion can be applied. By incorporating contrastive learning, the algorithm optimizes the feature space and improves the similarity representation between datasets. It learns to identify patterns and semantic similarities even in sparsely represented rare diseases.

The main contributions are summarized as follows:

(1) We augment the contrast samples by mixing sentence embeddings between positive pairs and propose the clustering model MCC, which is an extension of SCCL, and achieves good improvement in low resource cases.
(2) The contrastive loss is simplified to widen the similarity score difference between positive and negative pairs, leading to improved performance as demonstrated in our experiments.
(3) Through extensive comparative and ablation experiments on short text clustering tasks, we showcase the benefits of our approach. Not only do we reduce memory consumption and training time, but we also optimize the clustering score.

## 2 Related work

The core idea of this paper is to use contrastive learning to compare the similarity between samples to learn features, and then use clustering technology to cluster similar samples to discover hidden semantic structures. Mixup generates low-consumption virtual samples through linear interpolation to increase contrast samples. In this section, we will introduce the above three main concepts.

### 2.1 Short text clustering

Short text clustering is an important issue in text mining and aims to group short texts with similar topics or semantics into the same cluster. It is a crucial problem in natural language processing, information retrieval, social network analysis, and other fields. It roughly goes through two processes: Traditional clustering methods, such as K-means, hierarchical clustering, etc. The main advantage of these algorithms is that they are simple and easy to implement. However, due to the sparsity and high dimensionality of short texts, the performance of these algorithms is not very ideal for noisy data. Later, Singular Value Decomposition (SVD) (Golub and Reinsch, 1971), Principal Component Analysis (PCA) (Wold et al., 1987), and other methods extracted features from the data to reduce the dimension and noise of the data, retain more meaningful content, and improve the clustering effect. The other is the deep clustering method, which use deep neural networks (DNNS) to learn text features and clustering targets, have received extensive attention. DCN (Yang et al., 2017) combines autoencoder and traditional clustering, which makes features more discriminative and expressive in clustering, optimizes reconstruction loss and k-means loss, and has a simple objective and low complexity. DEC (Xie et al., 2016), the feature representation process and soft assignment of clustering are put together to jointly optimize the objective function. Although this deep clustering method does not surpass the most advanced clustering methods today, it lays a good foundation for later research. At the same time, the method based on deep learning can also take into account the semantic information of the text when dealing with short text clustering. Such methods are also adopted in our MCC, which combines clustering with deep representation learning and introduces contrastive learning to further learn discriminative representations.

### 2.2 Contrastive learning

Contrastive learning (CL) is a form of self-supervised learning that falls under the umbrella of unsupervised learning. Over the past 2 years, it has been extensively studied by researchers in the field. The main concept behind CL is to first construct positive and negative pairs through data augmentation, which pulls the distance between semantically similar samples and pushes the distance between semantically dissimilar samples. Measurement of the stability of feature representation is achieved through Alignment and Uniformity (Giorgi et al., 2020; Wang and Isola, 2020). Alignment ensures the features of positive pairs are as similar as possible, while uniformity ensures the distribution of features maintains as much information as possible. Early research efforts have focused on exploring various data augmentation strategies (Wu et al., 2020). SimCSE (Gao et al., 2021) introduces an implicit enhancement strategy, where positive pairs are constructed after applying two different dropout techniques. Consert (Yan et al., 2021) further investigates data augmentation methods, such as Cutoff and Token Shuffling, on feature matrices. Experimental results demonstrate the effectiveness of the implicit enhancement strategy. CC (Li et al., 2021) also incorporates a clustering objective, enabling contrastive learning at both the instance and cluster levels and leading to further optimization of the vector space. SCCL applies this method to text data and achieves state-of-the-art results. Additionally, the inclusion of contrast samples has been shown to improve performance. Therefore, MoCo utilizes a queue to store past small batches of datasets, increasing the availability of comparison data. It also proposes an encoder in the momentum update queue, which promotes a smooth transition in the high-dimensional space even when the input data is slightly altered or perturbed. ESimCSE (Wu et al., 2021) adopts a similar approach and employs word repetition as an enhancement method to address the limitation of GPU memory when expanding the batch size. However, it should be noted that this method requires calculating the distance between two network outputs at different time steps, which can prolong the training time. Exploring the learning of sentence representations in low-resource settings still requires further investigation.

In response, we propose combining mixup with SCCL. This approach involves summing the vector weights of the original and augmented data to generate a low-consumption virtual representation. To maintain semantic coherence, weights are biased more toward the feature representation of the original samples. The virtual representations are then optimized in an end-to-end manner. Our experiments demonstrate that the proposed approach enables the use of smaller batches during training while still achieving superior clustering scores. Additionally, this approach effectively alleviates the problems associated with GPU memory consumption and time occupation.

### 2.3 Mixup

Mixup is a virtual and implicit data augmentation technique that involves combining two different samples to generate a new training sample, with the goal of increasing the size and complexity of the dataset to improve the generalization ability and robustness of the model. It was initially used in image classification and works by linearly interpolating the input data and corresponding labels, overlapping the two images to create a new one. Mixup can be thought of as creating an infinite partition of the input space, which smooths the regularization of the model and reduces overfitting. Several variants and extensions have been developed, including CutMix (Yun et al., 2019), Puzzle Mix (Kim et al., 2020), and FMxi (Harris et al., 2020). Guo et al. (2019) have independently explored the mixture of the word embedding layer and the representation layer in text data and have substantiated its effectiveness through extensive comparative experiments. On the other hand, Manifold Mixup (Verma et al., 2019) introduces a novel approach by replacing the conventional input data mixing with the mixing of intermediate hidden layer outputs. This technique investigates the impact of mixing operations on each hidden layer embedded within the model. Mixup-transformer proposed using mixup on the transformer architecture to increase data diversity and generalization performance, with smaller datasets benefitting most from this augmentation. SSMix (Yoon et al., 2021) differs from other mixup methods that focus on hidden layers as it replaces some tokens in the text input while retaining most important tokens.

Overall, mixup introduces innovative ideas and techniques to the training and application of deep learning models. In our proposed model, we incorporate the concept of mixup by adding the vector weights of the original and augmented data. This process generates a virtual representation that requires lower computational resources. The weights are optimized in an end-to-end manner, aiming to bias them more toward the feature representation of the original sample. This strategy helps to preserve the semantics of the data during training. Remarkably, extensive experiments demonstrate that utilizing a smaller batch size for training can yield superior clustering scores while effectively mitigating the issues of GPU memory consumption and time occupation.

## 3 Model

**FIGURE 2:**
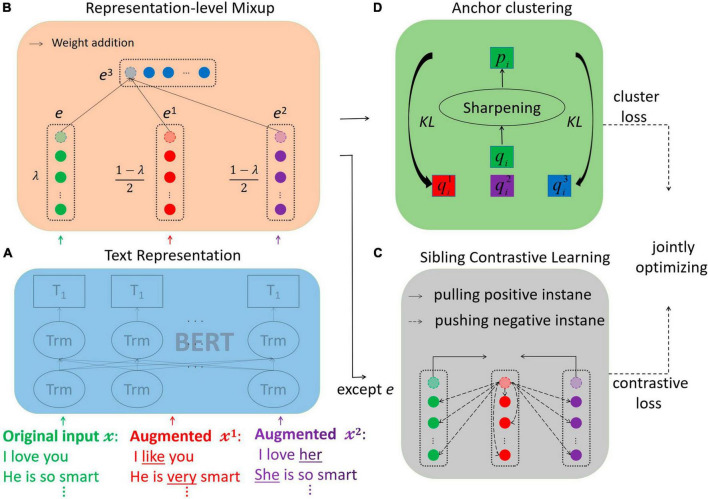
The MCC model. **(A)** Extracting text features using BERT. **(B)** Linearly combine the vectors of positive pairs. **(C)** Adjusting the distance between positive instances in the feature space. **(D)** Fine-tuning the clustering probability of the augmented text with KL scatter, and finally, jointly optimizing the clustering and comparison losses.

The goal of our proposed Model for Compositional Clustering is to facilitate low-resource representation learning by mixing to generate low-consumption representations. MCC consists of four main modules: the text representation module, the representation-level mixup module, the sibling contrastive learning module, and the anchor clustering module, as illustrated in Figure 2. (a) The text representation module is responsible for mapping the original text and two sets of augmented text into a low-dimensional feature vector using a pre-trained model. (b) The representation-level mixup module aims to mix and weight the representations of three sets of positive pairs, aligning them at corresponding index positions to create a set of virtual vectors. (c) The sibling contrastive learning module focuses on progressively reducing the distance between positive pairs from the two groups in the feature space, while pushing negative pairs away from positive pairs of the other group. (d) The anchor clustering module calculates the soft assignment probability of each sample to each cluster. The original dataset serves as the anchor point, and the soft assignment probability of the augmented dataset is optimized using KL divergence toward the target assignment probability. This process enhances the confidence of the clustering results. By leveraging the hybrid virtual dataset generated through these modules, the MCC model effectively captures more informative text patterns in scenarios with limited resources. Algorithm 1 provides a summary of the training and testing processes of the model in pseudo-code.

**Algorithm 1:** Training of MCC.

**Input:** datasets X; training epochs L; batch size N; temperature parameter τ; learning rate *Lr*; cluster number *K*; augmentations *U*^1^, *U*^2^; mixup weights λ **Output:** cluster assignments 1: initialization cluster center μ_*k*_ by K-means 2: **for** epochs = 1 to L **do** 3: obtain mini-batch of features from dataset X 4: mixing feature *E*_3_ by Eq. (1) 5: compute contrastive loss ℒ_*predict*−*ins*_ by Eq. (4) 6: compute cluster soft-assignments *q*_*ik*_, *p*_*ik*_ by Eq. (5) and Eq. (6) 7: compute cluster loss ℒ_*anchor*−*clu*_ by Eq. (7) 8: compute total loss ℒ by Eq. (8) 9: update *μ_*k*_* by minimize ℒ 10: **end for**

### 3.1 Text representation

Data augmentation serves as the initial stage of this module. Following recent research, we employ context augmentation (Kobayashi, 2018) as a means of data augmentation. This technique randomly masks words using a mask language model (MLM). Subsequently, the predicted words from the data augmentation model are utilized to replace the masked words, akin to synonym replacement. This approach effectively captures associations and semantic information between words. As data augmentation models, we choose BERT (Devlin et al., 2018) and Robert (Liu et al., 2019b), denoting their MLM encoders as *U*^1^ and *U*^2^, respectively. To begin, let us randomly select N samples and denote them as *M*_*i*_ = {*x*_1_, *x*_2_,, *x*_*N*_} from the original text X, with N representing the batch size and *i* serving as the subscript index. We then take the sampled *M_i_* and apply *U*^1^and *U*^2^ to generate two transformed data samples, resulting in $M_{i}^{1}=U^{1}\,\left(M_{i}\right)$ and $M_{i}^{2}=U^{2}\,\left(M_{i}\right)$, $M_{i}^{1}=\left\{x_{1}^{1},x_{2}^{1},,x_{N}^{1}\right\}$, $M_{i}^{2}=\left\{x_{1}^{2},x_{2}^{2},,x_{N}^{2}\right\}$. The samples corresponding to each position in *M*^1^and *M*^2^represent positive sample pairs, while samples from different positions represent negative sample pairs. For instance, $\left\{x_{1}^{1},x_{1}^{2}\right\}$ is a positive pair, whereas $\left\{x_{1}^{1},x_{1}^{2}\right\}$ is a negative pair. Subsequently, *M*_*i*,_ $M_{i}^{1}$, and $M_{i}^{2}$ are mapped to the feature space via an encoder, which yields the corresponding feature vectors *E*_*i*_={*e*_1_, *e*_2_,, *e*_*N*_}, $E_{i}^{1}=\left\{e_{1}^{1},e_{2}^{1},,e_{N}^{1}\right\}$, and $E_{i}^{2}=\left\{e_{1}^{2},e_{2}^{2},,e_{N}^{2}\right\}$. For the encoder backbone, in theory, our approach is not constrained to a specific neural network. In this paper, we simply choose distilbert-basenli-stsb-mean-tokens from the sentence-bert (Reimers and Gurevych, 2019) library as the basis for extracting feature vectors. The description in Algorithm 1 is line 3.

### 3.2 Representation-level mixup

Mixup is an implicit method to enhance the representation layer by performing linear interpolation, aiming to expand prior knowledge and improve model fitting ability and robustness.


(1) $$\begin{array}{c} e_{i}^{3}=\varphi \,\left(\lambda ,e_{i},e_{i}^{1},e_{i}^{2}\right)\\ =\lambda \,e_{i}+\left(\frac{1-\lambda }{2}\right)\,e_{i}^{1}+\left(\frac{1-\lambda }{2}\right)\,e_{i}^{2} \end{array}$$

Here φ(⋅) is the mixup function, $e_{i}^{3}$ is the feature vector generated after mixup, and $E_{i}^{3}=\left\{e_{1}^{3},e_{2}^{3},,e_{N}^{3}\right\}$ is obtained finally. λis the weight that determines the resulting sentence vector by adjusting ë, which can be a fixed value belonging to [0,1] or subject to *Beta*(α, α), αϵ(0).

Mixup increases the dataset size through linear interpolation of sentence vectors between positive samples, which also generates additional negative samples from different positions. By expanding the dataset size, mixup provides more data information to the model and improves training quality. Furthermore, mixup can reduce GPU memory consumption and training time compared to other data expansion techniques due to the smaller batch sizes needed to train on larger datasets. This process is described in Algorithm 1, with line 4 denoting the representation-level mixup that generates a new set of features by linearly interpolating between three sets of feature vectors at corresponding index positions.

### 3.3 Sibling contrastive learning

Narrowing the distance between two positive instances while extending the distance between a third positive instance and the negative instances in different groups. This method is referred to as sibling contrastive learning.

Positive sample pairs $\left\{e_{i}^{1},e_{i}^{2}\right\}$, $\left\{e_{i}^{2},e_{i}^{3}\right\}$, and $\left\{e_{i}^{3},e_{i}^{1}\right\}$ are formed by selecting samples from the same index positions in the augmented sample sets. Similarly, negative sample pairs $\left\{e_{i}^{k},e_{j}^{k}\right\}$are created by choosing samples from different index positions, where I, ∈ {1, 2,, *N*} represent the index, and i ≠ j, *k* ∈ {1, 2, 3} represent the label of the datasets so that 3N-3 negative pairs can be obtained. The vector $e_{i}^{k}$ is fed into a Multilayer Perceptron (MLP) architecture that consists of two fully connected layers with a ReLU activation function. The purpose of utilizing this MLP is to effectively reduce the dimensionality of the vector and perform normalization. As a result of this process, the output $z_{i}^{k}$ is obtained, reflecting the transformed and normalized representation of the original vector. We integrate them into the contrastive loss based InfoNCE, as shown in the following equation:


(2) $$\begin{array}{c} \ell_{i}^{1,2}=-\frac{1}{2}(log\frac{\exp\left(\text{ù}\,\left(z_{i}^{1},z_{i}^{2}\right)/\text{ô}\right)}{\sum_{j=1}^{N}\sum_{k=1}^{3}\left[\exp\left(\text{ù}\left(z_{i}^{1},z_{j}^{k}\right)/\text{ô}\right]\right]}+\\ log\frac{e\,x\,p\,\left(\text{ù}\,\left(z_{i}^{2},z_{i}^{1}\right)/\text{ô}\right)}{\sum_{j=1}^{N}\sum_{k=1}^{3}\left[exp\left(\text{ù}\left(z_{i}^{2},z_{j}^{k}\right)/\text{ô}\right]\right]}) \end{array}$$

Here, ù(⋅) is used to calculate the cosine distance between samples, the numerator represents the similarity of positive pairs, and the denominator represents the similarity of negative pairs. τ is a temperature coefficient that controls how well the model can distinguish between negative samples. In addition, we simplify the loss and propose a sibling contrastive learning method as follows:


(3) $$\ell_{i}^{1,2}=\left(z_{i}^{1},z_{i}^{2}|z_{i}^{3}\right)=-l\,o\,g\,\frac{\exp\left(\text{ù}\,\left(z_{i}^{1},z_{i}^{2}\right)/\text{ô}\right)}{\sum_{j=1}^{N}\sum_{k=1}^{3}\left[\exp\left(\text{ù}\,\left(z_{i}^{3},z_{j}^{k}\right)/\text{ô}\right)\right]}$$

In this method, we modify Equation (2) by replacing the anchor in the denominator with its sibling instance that shares the same attribute. By separating the anchor node from the negative instance and aggregating its sibling instance, we achieve the desired comparison effect. The description in Algorithm 1 is given as line 5. We aim to identify the positive instances in set $\left\{E_{i}^{1},E_{i}^{2},E_{i}^{3}\right\}$. Therefore, we compute the contrastive loss for each of the three data sets as follows:


(4) $${ℒ}_{p\,r\,e\,d\,i\,c\,t-i\,n\,s}=\frac{1}{3\,N}\,\sum_{i=1}^{N}\left(\ell_{i}^{1,2}+\ell_{i}^{1,3}+\ell_{i}^{2,3}\right)$$

### 3.4 Anchor clustering

The objective of this module is to classify the samples in the datasets and cluster similar samples together. Any sample *e_i_* in the original dataset *E_i_* is considered as an anchor. The positive samples $\left\{e_{i}^{1},e_{i}^{2},e_{i}^{3}\right\}$ in the three augmented datasets are treated as child nodes, ensuring that the cluster assignment of the anchor is consistent with that of the child nodes. This helps to achieve more accurate clustering.

Specifically, we use K-means to initial *K* cluster centers, defined as μ_*k*_, symbolizing the centroid of each cluster, to partition the samples into the nearest ì_*k*_, *k* ∈ {1, 2,, *K*}. The distance between sample *x_i_* and ì_*k*_ is measured by the soft assignment probability *q*_*ik*_ obtained by the Student’s t-distribution, where *q*_*ik*_ represents the probability that sample *x_i_* is assigned to the kth cluster center, as shown in the following equation:


(5) $$q_{i\,k}=\frac{{\left(1+{||e_{i}-{\text{ì}}_{k}||}_{2}^{2}/\alpha \right)}^{-\frac{\alpha +1}{2}}}{\sum_{k^{\prime }=1}^{K}{\left(1+{||e_{i}-{\text{ì}}_{k^{\prime }}||}_{2}^{2}/\alpha \right)}^{-\frac{\alpha +1}{2}}}$$

Here α represents the degree of freedom of the Student’s t-distribution; the larger α is, the closer the t-distribution curve is to the standard normal distribution; the smaller α is, the flatter the t-distribution curve is. Assigning samples to each of the *k* cluster centers results in a probability vector *q_i_* consisting of *q*_*ik*_, *q*_*i*_=[*q*_*i*1_, *q*_*i*2_,, *q*_*iK*_]. In order to focus on the data with higher confidence, the soft cluster assignment probability is raised to the second power to obtain the assistant probability *p*_*ik*_, which is expressed as follows:


(6) $$p_{i\,k}=\frac{q_{i\,k}^{2}/f_{k}}{\sum_{k^{\prime }}q_{i\,k}^{2}/f_{k^{\prime }}}$$

In this context, *f*_*k*_ = ∑_*i*_*q*_*ik*_ can be considered as an approximation that all samples in mini-batch N belong to the kth cluster, *k* ∈ {1, 2,, *K*}. Then, we normalize the soft assignment distribution to the second power to further improve confidence and reduce deviation caused by clustering imbalance. To achieve this transition from the soft assignment distribution to the assistant assignment distribution, we use KL divergence. The definition of KL divergence is as follows:


(7) $${ℒ}_{a\,n\,c\,h\,o\,r-c\,l\,u}=\frac{1}{3}\left(KL\left[p_{i}||q_{i}^{1}\right]+KL\left[p_{i}||q_{i}^{2}\right]+KL\left[p_{i}||q_{i}^{3}\right]\right)$$

Here, *p_i_* is the assistant assignment distribution obtained by the anchor in the original dataset through Equation (6), and $q_{i}^{1}$, $q_{i}^{2}$, and $q_{i}^{3}$ represent the soft assignment distribution obtained by the child nodes corresponding to the anchor in the three enhanced datasets through Equation (5). By optimizing the loss function of anchor clustering, we ensure that the child nodes continue to learn the features in the anchor points with high confidence. Moreover, it also helps the positive samples to come closer to each other. The detailed description of Algorithm 1 is provided in line 7.

### 3.5 Objective loss function

Our total loss can be summarized as follows:


(8) $$ℒ={ℒ}_{p\,r\,e\,d\,i\,c\,t-i\,n\,s}+\gamma \,{ℒ}_{a\,n\,c\,h\,o\,r-c\,l\,u}$$

The total loss is a combination of the contrastive loss and the clustering loss, where γ plays a crucial role in balancing the two losses. The detailed description of Algorithm 1 for this process is presented in line 8. The experiment is flexible, and it is possible to replace the clustering module loss by optimizing only the anchor assignment probability or only the child node assignment probability. Additionally, we plan to conduct comparative experiments comparing Equations (2) and (3) in the CL module, which we will explore in detail in the next chapter.

## 4 Experiment

To demonstrate the improvement brought about by adding mixup to the text vector space, we conducted extensive experiments on eight short text datasets. This chapter sequentially introduces the dataset selection, experimental settings, evaluation metrics, comparative experiments with baseline models, and ablation experiments. Finally, we will test the loss functions discussed in the previous chapter to evaluate their performance in our experiments.

## 5 Dataset

This study uses a dataset consisting of eight short text datasets, each with various data types, such as news headlines, article titles, and web search snippets. The dataset includes corresponding category labels. The following datasets were used: AgNews, StackOverflow, Biomedical, SearchSnippets, Googlenews-TS, Googlenews-S, Googlenews-T, and Tweet. The datasets presented offer a wide range of contexts and formats, increasing the potential applicability of the study to real-world situations. Table 1 provides a concise overview of each dataset, including their unique characteristics and the specific types of text data they contain.

**TABLE 1:** Overview of the dataset.

Dataset	|*V*|	Documents		Clusters	
		*N^D^*	Len	*N^C^*	L/S
AgNews (AN)	21K	8,000	23	4	1
SearchSnippets (SS)	31K	12,340	18	8	7
Biomedical (Bio)	19K	20,000	13	20	1
StackOverflow (SO)	15K	20,000	8	20	1
Tweet	5K	2,472	8	89	249
Googlenews-T (GT)	8K	11,109	6	152	143
Googlenews-S (GS)	18K	11,109	22	152	143
Googlenews-TS (GTS)	20K	11,109	28	152	143

## 6 Setup

In this paper, we employ context augmentation, word deletion, and random char as data augmentation methods, and Bert-base and Roberta as augmentation models. For the encoder, we use distilbert-base-nli-stsbmean-tokens to map the text to a feature space, with a maximum input length of 32. We run 2000 model iterations, using Adam as the optimizer with a learning rate of 5e-6 for the encoder, and a learning rate of 5e-4 for the optimized anchor node clustering head and the sibling instance contrastive learning head. For biomedicines, we set α = 10, while for other datasets, we also set α = 1. We attach a 768*K linear layer to the end of the clustering head to model the cluster centers, where K represents the number of clusters. Additionally, an MLP (Van der Maaten and Hinton, 2008) is attached to the end of the CL head to map the feature vectors to a subspace of size 768*128. For the mixing head, we tested different values of λ from the range of 0 to 1 and determined that a mixing weight of λ = 0.8 achieved the best clustering effect. The remaining main parameters are set as follows: η = 10, τ = 0. 5. We analyzed the experiments presented in Table 2 to determine the optimal value of λ.

**TABLE 2:** Comparison of accuracy between datasets AgNews and SearchSnippets across 32 batches.

λ	0.1	0.2	0.3	0.4	0.5	0.6	0.7	0.8	0.9
AN	87.2	87.1	87.2	87.2	87.2	87.2	87.2	87.2	87.1
SS	85.0	84.3	84.1	84.4	84.8	84.8	85.6	86.0	85.8

## 7 Evaluation metrics

We evaluate our model’s performance using two widely used metrics in clustering tasks, Normalized Mutual Information (NMI) and Accuracy (ACC), where higher scores indicate better clustering results, with values ranging from 0 to 1. Additionally, we include running time and GPU memory footprint as supplementary metrics, with smaller values indicating better model performance. Finally, we use K-means to predict the cluster centers of the feature vectors passing through the clustering module and the CL module.

## 8 Comparison with the baseline

We conducted experiments on eight short text datasets to evaluate the performance of MCC in clustering tasks. Our results show that MCC achieves highly competitive performance compared to other state-of-the-art methods. To provide a more comprehensive evaluation, we selected several classical text clustering methods as baseline models, including BOW, TF-IDF, K-means, DEC, STCC (Xu et al., 2017), Self-Train (Hadifar et al., 2019), HAC-SD (Rakib et al., 2020), and SCCL. The experimental results are the average of five experiments, and we obtained the results of the baseline models from their respective papers. As observed in Table 3.

**TABLE 3:** Experimental results on eight short text datasets.

Dataset	AgNews		SearchSnippets		Biomedical		StackOverflow	
Metrics	NMI	ACC	NMI	ACC	NMI	ACC	NMI	ACC
BOW	2.6	27.6	9.3	24.3	9.2	14.3	14.0	18.5
TF-IDF	11.9	34.5	19.2	31.5	23.2	28.3	58.7	58.4
K-means	59.2	83.9	36.4	59.0	32.7	39.8	52.3	60.8
DEC	–	–	64.9	76.9	37.7	41.6	75.3	74.7
STCC	–	–	63.2	77.0	38.1	43.6	54.8	59.8
Self-Train	–	–	56.7	77.1	47.1	54.8	64.8	64.8
HAC-SD	54.6	82.8	63.8	82.7	33.5	40.1	59.5	64.8
SCCL	**68.2**	**88.2**	71.1	85.2	41.5	46.2	74.5	75.5
MCC	67	87.2	**71.9**	**86.0**	**42.3**	**49.1**	**76.1**	**77.6**
**Dataset**	**Tweet**		**Googlenews-T**		**Googlenews-S**		**Googlenews-TS**	
**Metrics**	**NMI**	**ACC**	**NMI**	**ACC**	**NMI**	**ACC**	**NMI**	**ACC**
BOW	73.6	49.7	73.2	49.8	73.5	49.0	81.9	57.5
TF-IDF	80.7	57.0	79.3	58.9	83.0	61.9	88.9	68.0
K-means	79.0	51.7	83.3	62.2	87.5	67.8	78.4	56.0
DEC	–	–	–	–	–	–	–	–
STCC	–	–	–	–	–	–	–	–
Self-Train	–	–	–	–	–	–	–	–
HAC-SD	85.2	89.6	84.2	81.8	83.5	80.6	88.0	85.8
SCCL	89.2	78.2	**88.3**	75.8	**90.4**	**83.1**	**94.9**	**89.8**
MCC	**89.6**	**79.5**	88.1	**76.3**	90.1	83.0	94.6	89.4

Bold values represent optimal values for the experimental setting.

**FIGURE 3:**
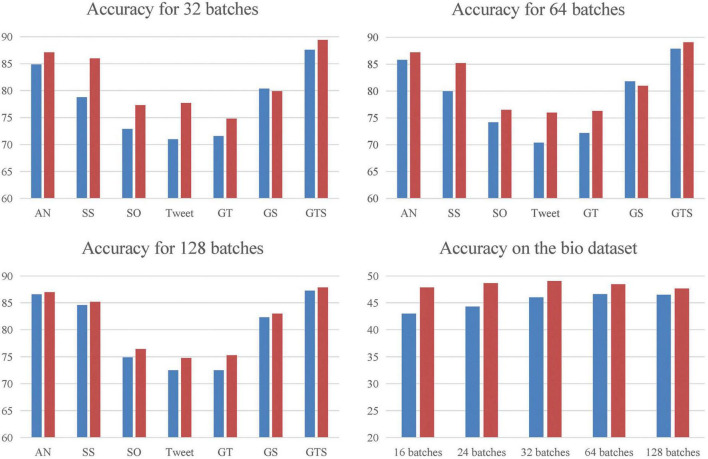
We compared MCC with the baseline in five low-quality environments, with data batch sizes set to 16, 24, 32, 64, and 128, respectively, and the results are shown in separate plots. To alleviate the score interval difference between Biomedical dataset and other datasets, a separate plot is made for it.

We used two experimental environments: one with a data batch size set to 400, and another with lower data batch sizes ranging from 16 to 128. In the first experiment, the evaluation metrics of our model outperform the baseline model on most datasets, particularly on StackOverflow, Tweet, and Biomedical datasets, where ACC is improved by 2–3%, respectively. The results are shown in Figure 3. In the second experiment, when compared with SCCL under the same batches, our model shows a significant improvement over the baseline.

**FIGURE 4:**
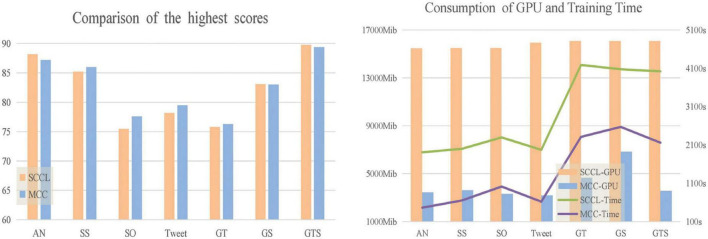
Comparison of time and GPU when MCC and SCCL get the highest score.

Furthermore, we compared the model training time and GPU memory footprint of MCC and SCCL when achieving the highest clustering score. As observed in Figure 4, our model has significantly reduced training time and memory footprint compared to the baseline, while also improving accuracy. In the contrastive learning phase, SCCL optimizes the data features by learning a large number of information features that enhance the data pairs. MCC employs mixup and sibling instance contrast to enlarge the sample size and contract the distribution between the original data and the positive pair, improving model performance.

As observed, the performance of biomedical datasets differs significantly from that of other datasets. We believe this is due to the limited amount of biomedical data in the corpus of the transformer pre-training model, leading to insufficient learning for such datasets and inaccurate feature information. Hence, our model brings significant improvement to biomedical datasets, enabling it to learn more prior knowledge about such data. However, biomedical datasets typically contain a significant amount of high-dimensional data, such as genes and proteins, which possess complex and variable characteristics that are difficult to generalize and classify. Additionally, they may contain a considerable amount of noise and outliers, which can interfere with the performance of clustering algorithms and result in a degradation of the clustering quality. There is still much room for improvement in the future.

## 9 Ablation experiment

Ablation experiments were conducted on the Tweet dataset to prove the effectiveness of each module in the model. We successively discarded parts of the loss to demonstrate the importance of each component to the model, as shown in Table 4. We used k-means behind the presentation layer to calculate the score performance in the three cases. The experiments demonstrated that mixup and anchor clustering are important components.

**TABLE 4:** Performance of each loss on the Tweet dataset.

LOSS	ACC	NMI
Cluster	73.4	85.1
Instance + Cluster	78.2	89.2
Instance + Cluster + Mixup	**79.5**	**89.6**

With the addition of a contrastive learning module, our model achieves better results. This is because the “same class is different” challenge is alleviated, and samples of the same class are close to each other in the feature space, while the samples of different classes are more scattered. Contrastive learning can improve the discriminative power and distinction of the clustering model by emphasizing the similarity between samples of the same class and the difference between samples of different classes. After introducing mixup, we can generate virtual samples, which have certain combination characteristics. The model can learn more relative relationships and boundaries between samples, generalize better to unseen samples, and focus on the correlation between samples in the learning process.

## 10 Comparative experiment

This section describes experiments conducted to select the best data augmentation methods and clustering loss to achieve optimal performance of the model. We conducted comparative experiments and analyzed the results to determine the most effective combination of methods and loss functions.

### 10.1 Performance of data augmentation

Through our research, we found that different data augmentation methods yield varying performances when used in our method. To verify the importance of data augmentation, we compared the performance of three augmentation methods on the model: word deletion, random char, and context augmentation. As shown in Table 5, these strategies produced different clustering scores.

**TABLE 5:** Performance of different data augmentation.

Dataset	Augmentation	ACC	NMI
AgNews	Random char	86.0	64.9
	Word deletion	87.1	67.2
	Context augmentation	**87.2**	**67.3**
SearchSnippets	Random char	84.6	70.5
	Word deletion	64.8	56.5
	Context augmentation	**86.0**	**71.9**
Biomedical	Random char	44.8	39.7
	Word deletion	45.1	40.2
	Context augmentation	**49.1**	**42.3**
StackOverflow	Random char	72.3	69.6
	Word deletion	74.9	**77.4**
	Context augmentation	**77.6**	76.1
Tweet	Random char	75.1	87.6
	Word deletion	77.6	88.5
	Context augmentation	**79.5**	**89.6**
Googlenews-T	Random char	73.4	86.5
	Word deletion	73.8	86.8
	Context augmentation	**76.3**	**88.1**
Googlenews-S	Random char	80.0	88.9
	Word deletion	81.9	89.3
	Context augmentation	**83.0**	**90.1**
Googlenews-TS	Random char	87.3	93.8
	Word deletion	88.1	94.0
	Context augmentation	**89.4**	**94.6**

Random char can enhance the model’s robustness to minor misalignments or spelling errors in the input, thereby improving its generalization ability. However, it is important to note that this technique may inadvertently alter the meaning or grammatical structure of words, potentially resulting in unnatural or semantically distorted text generation. On the other hand, word deletion serves as a means to simulate noise and missing information in textual data, thereby enhancing the model’s capacity to process incomplete text. Nevertheless, there is a risk of disrupting the intended meaning of the original text, leading to generated text that appears disjointed or semantically unclear. Context enhancement, which involves predicting blocked words to enrich the article’s information, introduces additional variations and diversities. This approach helps the model adapt more effectively to different contextual scenarios.

Overall, their effects are carefully balanced in order to preserve the coherence and meaning of the original text while enhancing the power of the model.

### 10.2 Performance of anchor loss function

The loss function of the clustering module is studied in the traditional contrastive module and the sibling contrastive module, respectively. Specifically, it is to prove the effectiveness of the selected function by replacing the anchor function with the potential objective function and then optimizing its loss function combined with Eq. (2) and (3). As shown in Table 6, pushing the augmentation to the anchor clustering module with the Sibling contrastive learning module leads to the highest score.

**TABLE 6:** Performance of different anchor loss functions on the tweet dataset.

Contrastive-head	Clustering-head	ACC	NMI
Eq. (3)	*KL*[*q*_*i*_, *p*_*i*_]	77.4	88.5
	$K\,L\,\left[q_{i}^{1},p_{i}^{1}\right]+K\,L\,\left[q_{i}^{2},p_{i}^{2}\right]+K\,L\,\left[q_{i}^{3},p_{i}^{3}\right]$	76.8	88.4
	$K\,L\,\left[q_{i},p_{i}^{1}\right]+K\,L\,\left[q_{i},p_{i}^{2}\right]+K\,L\,\left[q_{i},p_{i}^{3}\right]$	**79.2**	**89.4**
Eq. (4)	*KL*[*q*_*i*_, *p*_*i*_]	77.6	88.4
	$K\,L\,\left[q_{i}^{1},p_{i}^{1}\right]+K\,L\,\left[q_{i}^{2},p_{i}^{2}\right]+K\,L\,\left[q_{i}^{3},p_{i}^{3}\right]$	77.0	88.3
	$K\,L\,\left[q_{i},p_{i}^{1}\right]+K\,L\,\left[q_{i},p_{i}^{2}\right]+K\,L\,\left[q_{i},p_{i}^{3}\right]$	**79.5**	**89.6**

## 11 Conclusion

In this article, we aim to model scarce rare disease data using small batches of data. Our proposed hybrid-based short-text clustering comparison learning algorithm introduces hybridisation in the feature extraction phase. In small batch experiments on eight short-text datasets, our proposed algorithm concentrates on constructing features and expanding the comparison samples with minimal computational pressure. As a result, we achieved effective improvements across multiple datasets. For the short-text clustering issue in the medical sector, our algorithm can reduce GPU memory consumption and training time, besides providing more precise clustering outcomes. This method offers a practical solution to process medical text data for healthcare professionals, proficiently advancing medical technology. In the future, we plan to improve our algorithm and test it on larger datasets. We will also explore other hybridization techniques to enhance the feature extraction phase. Furthermore, we aim to integrate our algorithm into medical technology systems for real-time processing and disease prediction.

## Data availability statement

Publicly available datasets were analyzed in this study. This data can be found here: https://github.com/rashadulrakib/short-text-clustering-enhancement/tree/master/data.

## Author contributions

QX: Writing—review and editing. HZ: Writing—original draft. SJ: Writing—review and editing.
